# Long-acting injectable paliperidone palmitate versus oral paliperidone extended release: a comparative analysis from two placebo-controlled relapse prevention studies

**DOI:** 10.1186/1744-859X-12-22

**Published:** 2013-07-11

**Authors:** Michael Markowitz, Dong-Jing Fu, Bennett Levitan, Srihari Gopal, Ibrahim Turkoz, Larry Alphs

**Affiliations:** 1 Janssen Scientific Affairs, LLC, 1125 Trenton-Harbourton Road, Titusville, NJ 08560, USA; 2 Janssen Research & Development, LLC, 1125 Trenton-Harbourton Road, Titusville, NJ 08560, USA

**Keywords:** *Post hoc* analysis, Paliperidone extended release, Long-acting injectable, Paliperidone palmitate

## Abstract

**Background:**

Increasing availability and use of long-acting injectable antipsychotics have generated a need to compare these formulations with their oral equivalents; however, a paucity of relevant data is available.

**Methods:**

This *post hoc* comparison of the long-term efficacy, safety and tolerability of maintenance treatment with paliperidone palmitate (PP) versus oral paliperidone extended release (ER) used data from two similarly designed, randomised, double-blind (DB), placebo-controlled schizophrenia relapse prevention trials. Assessments included measures of time to relapse, symptom changes/functioning and treatment-emergent adverse events (TEAEs). Time to relapse between treatment groups was evaluated using a Cox proportional hazards model. Between-group differences for continuous variables for change scores during the DB phase were assessed using analysis of co-variance models. Categorical variables were evaluated using Chi-square and Fisher's exact tests. No adjustment was made for multiplicity.

**Results:**

Approximately 45% of enrolled subjects in both trials were stabilised and randomised to the DB relapse prevention phase. Risk of relapse was higher in subjects treated with paliperidone ER than in those treated with PP [paliperidone ER/PP hazard ratio (HR), 2.52; 95% confidence interval (CI), 1.46–4.35; *p* < 0.001]. Similarly, risk of relapse after withdrawal of paliperidone ER treatment (placebo group of the paliperidone ER study) was higher than after withdrawal of PP (paliperidone ER placebo/PP placebo HR, 2.25; 95% CI, 1.59–3.18; *p* < 0.001). Stabilised schizophrenic subjects treated with PP maintained functioning demonstrated by the same proportions of subjects with mild to no difficulties in functioning at DB baseline and end point [Personal and Social Performance (PSP) scale total score >70, both approximately 58.5%; *p* = 1.000] compared with a 10.9% decrease for paliperidone ER (58.5% vs 47.6%, respectively; *p* = 0.048). The least squares mean change for Positive and Negative Syndrome Scale (PANSS) total score at DB end point in these previously stabilised subjects was 3.5 points in favour of PP (6.0 vs 2.5; *p* = 0.025). The rates of TEAEs and AEs of interest appeared similar.

**Conclusions:**

This analysis supports maintenance of effect with the injectable compared with the oral formulation of paliperidone in patients with schizophrenia. The safety profile of PP was similar to that of paliperidone ER. Future studies are needed to confirm these findings.

## Background

Long-term maintenance therapy in schizophrenia is an important clinical and public health concern requiring careful balance between benefits and risks. This problem is important on both an individual patient level and a societal public health level, as the medical and societal costs of relapsing patients are high [1].

A major obstacle to the effective treatment of patients with schizophrenia is non-adherence with the medication regimen. It is estimated that 1-year rates of treatment discontinuation or interruption range from 40% to 75% [2,3]. Individual patients may discontinue or interrupt treatment as the result of a variety of factors, such as lack of illness insight, forgetfulness, lack of social support, tolerability issues, conscious choice and refractory or non-responsive symptoms [4,5]. Long-acting injectable (LAI) medications can help overcome problems with non-adherence by removing the need for daily dosing and by simplifying treatment; additionally, the healthcare provider can know with certainty whether a patient has received an injection and can make an appropriate intervention if the patient has not [6]. Several studies have shown that switching from an oral to an LAI antipsychotic is both safe and effective [7-10]. Unfortunately, the few head-to-head studies that have compared oral and injectable antipsychotics in terms of safety and efficacy have design limitations such as a short duration of treatment or a small sample size [11,12]. Long-term head-to-head comparisons against alternative oral treatment approaches have not been done [7-10]. To provide some basis for comparing the formulations, we conducted a *post hoc* indirect comparative efficacy analysis aimed at comparing long-term efficacy, including relapse prevention, and safety/tolerability of once-monthly paliperidone palmitate (PP) against oral paliperidone extended release (ER) using indirect comparative techniques and subject-level data from two similarly designed maintenance treatment studies [13,14].

## Methods

### Indirect comparison

In the absence of head-to-head trials comparing the long-term efficacy and safety of ER and PP, patient-level data on maintenance treatment from two similarly designed relapse studies were used [13,14] to conduct an indirect comparison for this *post hoc* analysis. Meta-analysis of relapse studies of other antipsychotics in the literature was not pursued because of widely varying study criteria and differences in the definition of *relapse*. It is known that differences in the definition of relapse significantly affect identified relapse rates [15]. The standard approach for an indirect comparison accounts for some difference between trials by placebo adjustment. However, because the LAI stays in the bloodstream for months compared with days for the oral ER formulation [16], the group that is withdrawn from PP and given a placebo still has active treatment in their bloodstream for several months [terminal half-life (t_½_), 25 to 49 days, depending on the dose] [17], while the group given an oral placebo has no substantial active treatment after 4 to 5 days (t_½_ = 23 h). Placebo-corrected comparisons therefore are not meaningful in this randomised withdrawal setting because of the dramatically different pharmacokinetics of the formulations. For this work, we have compared active with active and placebo with placebo directly. As discussed later, this approach is supported by similarity of design and baseline patient properties in the trials selected.

### Identification of source data

**Figure 1:**
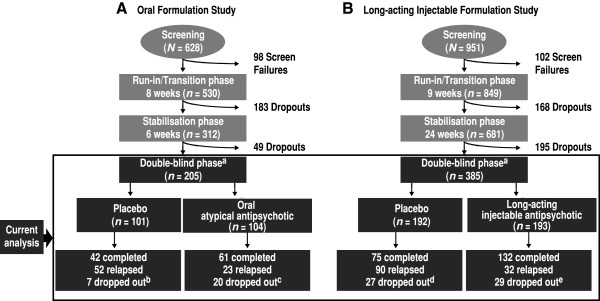
**Designs of the two studies used in the analysis. A** Paliperidone extended release (ER) vs placebo study. **B** Paliperidone palmitate (PP) vs placebo study. ^a^Subjects remained in the double-blind phase until they experienced a relapse, until they withdrew from the study or until the study was completed. The double-blind phase was followed by an open-label extension in each study. Based on significant efficacy, both studies were terminated early. At the time the studies were stopped, a total of 91 subjects in the paliperidone ER study and a total of 76 subjects in the PP study were in the stabilisation phase and were considered as completing the entire study per protocol. Subjects (*n* = 25) in the PP study who did not meet the more stringent stabilisation criteria employed in the paliperidone ER study were excluded to standardise the study criteria. ^b^Seven dropouts [1 adverse event (AE), 1 death, 2 lost to follow-up (FU) and 3 other]. ^c^Twenty dropouts (12 withdrew consent, 3 AEs, 2 lost to FU, 1 study drug protocol violation and 2 other). ^d^Twenty-seven dropouts (15 withdrew consent, 2 AEs and 10 other). ^e^Twenty-nine dropouts (12 withdrew consent, 3 AEs and 14 other).

Two similarly designed randomised, double-blind (DB), placebo-controlled schizophrenia relapse prevention studies of paliperidone ER (NCT00086320) [13] and PP (NCT00111189) [14] were included (Figure 1). These trials had comparable study designs (i.e. run-in/transition, stabilisation, DB and optional open-label extension phases), comparable stabilisation and relapse criteria and similar inclusion and exclusion criteria. The paliperidone ER study had an 8-week run-in phase followed by a 6-week open-label stabilisation phase [13], and the PP trial had a 9-week run-in phase followed by a 24-week open-label stabilisation phase [14]. In both trials, subjects were first stabilised with paliperidone ER or PP [defined by Positive and Negative Syndrome Scale (PANSS) total scores ≤75 and select PANSS item scores ≤4] and were then randomly assigned to active treatment or placebo. The DB phase was variable in the two trials: subjects remained in the DB phase until they experienced a relapse, until they withdrew from the study or until the study was completed. Therefore, the length of time in the DB period differed for each patient. Based on significant improvements over placebo, both studies were terminated early. Analysis was performed on the DB relapse prevention phase of the two studies, using the DB intent-to-treat analysis set, consisting of all randomly assigned subjects who received at least one dose of DB medication. Both original studies were conducted in accordance with the ethical principles that have their origin in the Declaration of Helsinki and that are consistent with Good Clinical Practices and applicable regulatory requirements. The original study protocols were reviewed and approved by an independent ethics committee or an institutional review board at each study site, and all subjects provided written informed consent before entering the studies.

The two studies had similar but slightly different stabilisation criteria. To match their randomisation in this analysis, subjects (*n* = 25) were excluded from the PP study because they did not meet the more stringent measures of PANSS ≤70 and Clinical Global Impressions-Severity (CGI-S) ≤4 (not ill to moderately ill), which were employed as stabilisation criteria in the oral formulation study.

### Definition of outcomes

This *post hoc* analysis was performed on efficacy and safety measures during the DB phase of the two studies.

Relapse during the clinical trials was defined as the first occurrence of one or more of the following:

• Psychiatric hospitalisation (involuntary or voluntary admission)

• Increase of 25% in PANSS total score for two consecutive assessments for subjects who scored >40 at randomisation, or a 10-point increase for subjects who scored ≤40 at randomisation

• Deliberate self-injury or aggressive behaviour, or suicidal or homicidal ideation and aggressive behaviour that was clinically significant

• Increase for two consecutive assessments in pre-specified individual PANSS item scores (P1, P2, P3, P6, P7 and G8) to ≥5 for subjects whose score was ≤3 at randomisation, or to ≥6 for subjects whose score was 4 at randomisation

In the paliperidone ER study only [13], relapse was also defined as an increase in CGI-S score to ≥4 for subjects who scored ≤3 at randomisation, or to ≥5 for subjects whose score was 4 at randomisation for two consecutive assessments. Subjects who met the CGI-S criterion also met the other relapse criteria; therefore, no adjustment in relapse definition was necessary for this analysis. Definitions of maintenance phase outcomes, other than relapse, are shown in Table 1.

**Table 1:** Efficacy and safety outcomes

**Outcome**	**Measures and definitions**^**a**^
Efficacy	
CGI-S	Change in CGI-S score
PANSS	Change in total PANSS scores
Relapse	Time to first relapse and percentage relapse
PSP	Change in PSP total score
	Frequency of PSP category change (categories 0 to 70, 71 to 100)
Safety	
AEs	Overall incidence, *n* (%)
	Serious AEs, *n* (%)
	AEs leading to discontinuation, *n* (%)
	AEs of interest (EPS-related and prolactin-related AEs), *n* (%)
Weight gain	≥7% weight increase

^a^From double-blind baseline to double-blind end point. *AE* adverse event, *CGI-S* Clinical Global Impressions–Severity, *EPS* extrapyramidal symptoms, *PANSS* Positive and Negative Syndrome Scale, *PSP* Personal and Social Performance.

### Statistical methods

Demographic and baseline characteristics were summarised using descriptive statistics for the DB phase of each study. The baseline demographic and disease characteristics of both studies were also compared to identify potential confounders. Time to relapse between treatment groups was evaluated using Cox proportional hazards analysis and the log-rank test. The cumulative distribution function of the time to relapse was estimated by the Kaplan-Meier method. Between-group differences for continuous variables for change scores during the DB phase were assessed using analysis of co-variance models with baseline values as the continuous co-variate and using the last observation carried forward method for imputation of missing values. Categorical variables were evaluated using odds ratios, Chi-square tests and Fisher's exact test. Shifts from baseline to end point in Personal and Social Performance (PSP) categories were examined using McNemar's test. Treatment-emergent adverse event (TEAE) rates were also compared. No adjustment was made for multiplicity.

## Results

Baseline demographic and clinical characteristics of the two study populations appeared comparable except for race because the studies were conducted in different countries (Table 2). A total of 385 subjects were included in the PP study (193 PP, 192 placebo), and 205 in the paliperidone ER study (104 paliperidone ER, 101 placebo). Table 3 summarises the extent of exposure of the two studies. As described in the original studies [13,14], both trials were stopped after interim analyses as a result of significant benefit of PP and ER over placebo. These early terminations resulted in median duration of exposure in the DB phase of 170 days (range 1 to 407 days) for the PP study versus 45 days (range 3 to 330 days) for the paliperidone ER study.

**Table 2:** Double-blind baseline demographics and clinical characteristics

	**Study active arms**		**Study placebo arms**	
	**PP**	**Paliperidone ER**	**PP placebo**	**Paliperidone ER placebo**
	**(*n *= 193)**	**(*n *= 104)**	**(*n *= 192)**	**(*n *= 101)**
Age in years, mean (SD)	38.6 (11.4)	39.0 (10.7)	39.2 (10.8)	37.5 (10.4)
Sex, *n* (%)				
Male	104 (53.9)	58 (55.8)	104 (54.2)	63 (62.4)
Female	89 (46.1)	46 (44.2)	88 (45.8)	38 (37.6)
Race, *n* (%)				
Caucasian	124 (64.3)	62 (59.6)	126 (65.6)	61 (60.4)
Black	36 (18.7)	8 (7.7)	35 (18.2)	9 (8.9)
Asian	30 (15.5)	3 (2.9)	27 (14.1)	0
Other	3 (1.6)	31 (29.8)^a^	4 (2.1)	31 (30.7)^a^
Age at diagnosis in years, mean (SD)	26.3 (9.2)	27.1 (9.2)	28.2 (8.9)	25.8 (9.4)
Baseline PANSS total score, mean (SD)	50.8 (11.0)	51.0 (11.4)	51.9 (11.1)	53.4 (10.6)
Baseline CGI-S score, *n* (%)				
Not ill	11 (5.7)	6 (5.8)	11 (5.7)	5 (5.0)
Very mildly ill	69 (35.8)	38 (36.5)	77 (40.1)	33 (32.7)
Mildly ill	92 (47.7)	49 (47.1)	81 (42.2)	54 (53.5)
Moderately ill	21 (10.9)	11 (10.6)	23 (12.0)	9 (8.9)
Previous hospitalisations for psychosis, mean (SD)	2.6 (1.2)^a^	2.9 (1.2)^b^	2.7 (1.2)^a^	2.9 (1.2)^c^

^a^*n* = 173; ^b^*n* = 78; ^c^*n* = 74. *CGI-S* Clinical Global Impressions–Severity, *ER* extended release, *PANSS* Positive and Negative Syndrome Scale, *PP* paliperidone palmitate, *SD* standard deviation.

**Table 3:** Extent of exposure

	**PP**	**Paliperidone ER**
Median duration, days (range)	170 (1 to 407)	45 (3 to 330)
Mean (SD) dose	128.0 (38.7) mg/month	10.8 (3.3) mg/day
Mode monthly/daily dose (percentage of subjects)	156 mg/month (64)	9 mg/day (38)

*ER* extended release, *PP* paliperidone palmitate.

### Efficacy

Patients were required to be stable (defined as PANSS total scores ≤75 and select PANSS item scores ≤4) to enter the DB period. Therefore, efficacy was measured by the degree to which this stability was lost, which was a result of relapse and corresponding efficacy and functionality findings.

**Figure 2:**
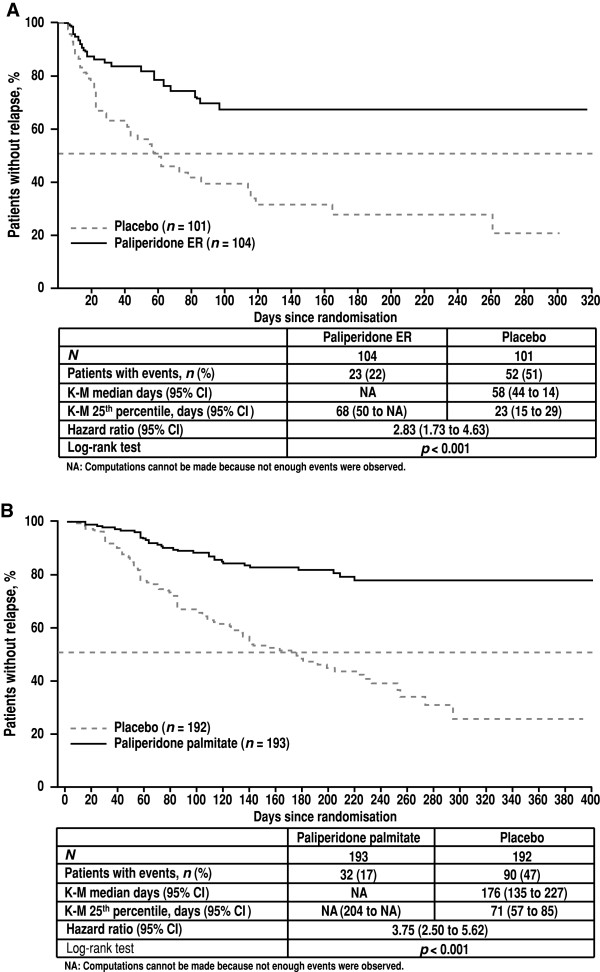
**Kaplan-Meier curves for relapse in treatment and placebo groups. (A)** Paliperidone extended release (ER). **(B)** Paliperidone palmitate. *CI* confidence interval, *ER* extended release, *K-M* Kaplan-Meier.

The two previously conducted clinical trials provide the basis for this *post hoc* comparison. Both showed active treatment to be superior to placebo in schizophrenia relapse studies (Figure 2) [13,14].

**Figure 3:**
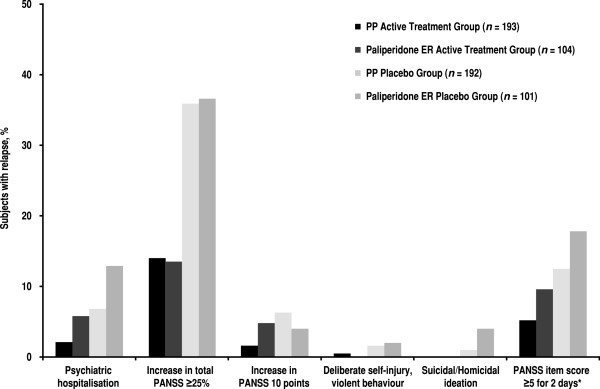
**Frequency of different types of relapse during double-blind phase (entire exposure period).** Categories are not mutually exclusive; some subjects met >1 relapse criterion. Asterisk indicates increase for two consecutive assessments in pre-specified individual PANSS item scores to ≥5 for subjects whose score was ≤3 at randomisation. *ER* extended release, *PANSS* Positive and Negative Syndrome Scale, *PP* paliperidone palmitate.

In this indirect comparative analysis of active treatment groups, the risk of relapse was higher in subjects treated with paliperidone ER than in subjects treated with PP (paliperidone ER/PP HR 2.52; 95% CI 1.46 to 4.35; *p* < 0.001) (Table 4). Similarly, the risk of relapse after withdrawal of paliperidone ER treatment (placebo group of the paliperidone ER study) was higher than after withdrawal of PP (paliperidone ER placebo/PP placebo HR 2.25; 95% CI 1.59 to 3.18; *p* < 0.0001). The most common reason for relapse was an increase in total PANSS of ≥25% (Figure 3).

**Table 4:** Indirect comparison of hazard ratios for risk of relapse

	**PP**	**Paliperidone ER**	**PP placebo**	**Paliperidone ER placebo**
	**(*n *= 193)**	**(*n *= 104)**	**(*n *= 192)**	**(*n *= 101)**
Subjects with events, *n* (%)	32 (17)	23 (22)	90 (47)	52 (51)
Hazard ratio (95% CI)^a^	2.52 (1.46 to 4.35)		2.25 (1.59 to 3.18)	

^a^Hazard ratios reflect paliperidone ER/PP for active treatments and placebo treatments, respectively. *CI* confidence interval, *ER* extended release, *PP* paliperidone palmitate.

**Figure 4:**
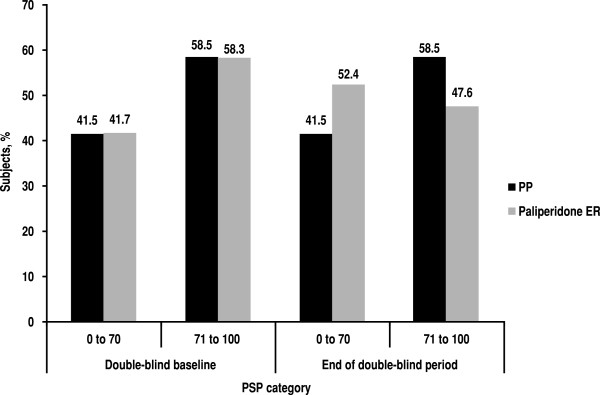
**Frequency of Personal and Social Performance (PSP) categories of score during double-blind phase.***ER* extended release, *PP* paliperidone palmitate.

Efficacy results for PANSS, PSP and CGI-S, comparing PP and paliperidone ER treatment, are shown in Table 5. During the DB phase, both PP and paliperidone ER treatment groups showed slight worsening in efficacy outcomes from baseline (i.e. increase in PANSS total scores and CGI-S scores) and in functioning outcomes (i.e. decrease in PSP total scores). The least squares (LS) mean change for the PANSS total score at DB end point (6.0 for the paliperidone ER study and 2.5 for the PP study) was 3.5 points in favour of PP and was statistically significant (*p* = 0.025), indicating that on average, subjects treated with PP demonstrated better maintenance of their DB baseline status than those treated with the oral formulation. This difference was evident, even though the period of observation for the LAI treatment group was longer than for the paliperidone ER treatment group (median 170 days for the PP study vs 45 days for paliperidone ER). No significant between-group differences were noted in the change in mean PSP or CGI-S scores from baseline. However, less decline in functional status was observed, as measured by PSP in subjects treated with PP (Figure 4); those treated with PP demonstrate virtually no difference between DB baseline and end point in the proportion of subjects with mild to no difficulties in functioning (PSP score >70; 58.5% vs 58.5%, respectively; *p* = 1.000), compared with a 10.9% decrease for paliperidone ER (58.3% vs 47.6%, respectively; *p* = 0.048).

**Table 5:** Efficacy outcomes from the double-blind period

		**Paliperidone**	***p *Value**
**Efficacy measure**	**PP**	**ER**	**(paliperidone ER vs PP)**
PANSS total score	*n* = 191	*n* = 104	
Double-blind baseline mean (SD)	50.8 (10.9)	51.0 (11.4)	
LS mean (SE) change from baseline to end point	2.5 (0.9)	6.0 (1.3)	0.025
PSP score	*n* = 188	*n* = 103	
Double-blind baseline mean (SD)	72.7 (10.5)	70.8 (10.9)	
LS mean (SE) change from baseline to end point	−1.4 (0.8)	−3.3 (1.1)	0.156
CGI-S score	*n* = 191	*n* = 104	
Double-blind baseline mean (SD)	2.6 (0.8)	2.6 (0.8)	
LS mean (SE) change from baseline to end point	0.2 (0.1)	0.3 (0.1)	0.095

*CGI-S* Clinical Global Impressions–Severity, *ER* extended release, *LS* least squares, *PANSS* Positive and Negative Syndrome Scale, *PP* paliperidone palmitate, *PSP* Personal and Social Performance, *SD* standard deviation, *SE* standard error.

### Safety

What appeared to be similar incidences of adverse events (AEs) of interest [extrapyramidal symptoms (EPS)-related AEs, prolactin-related AEs and weight increase] were observed (PP and paliperidone ER, respectively): low incidence of AEs leading to discontinuation (1.6% vs 2.9%); ≥1 serious treatment-emergent AE (4.2% vs 7.7%); EPS-related AEs (5.7% vs 6.7%); prolactin-related AEs (2.1% vs 2.9%) and weight increase ≥7% (23.4% vs 19.6%) (Table 6).

**Table 6:** Adverse events summary from the double-blind period

	**PP**	**Paliperidone ER**	**PP placebo**	**Paliperidone ER placebo**
	**(*n *= 193)**	**(*n *= 104)**	**(*n *= 192)**	**(*n *= 101)**
All TEAEs, *n* (%)	83 (43.0)	36 (34.6)	84 (43.8)	41 (40.6)
≥1 serious TEAE	8 (4.2)	8 (7.7)	23 (12.0)	16 (15.8)
TEAE leading to discontinuation	3 (1.6)	3 (2.9)	1 (0.5)	1 (1.0)
EPS-related AEs	11 (5.7)	7 (6.7)	3 (1.6)	3 (3.0)
Prolactin-related AEs	4 (2.1)	3 (2.9)	3 (1.6)	0
Weight increase ≥7%	44 (23.4)	19 (19.6)	24 (13.0)	11 (11.7)

*AE* adverse event, *EPS* extrapyramidal symptoms, *ER* extended release, *PP* paliperidone palmitate, *TEAE* treatment-emergent AE.

## Discussion

Results from the two previously published studies show that differences between treatment groups in time to relapse significantly favoured paliperidone ER and PP, compared with placebo. The indirect comparison between studies described here suggests that the risk of relapse was significantly higher in subjects treated with paliperidone ER as compared with subjects treated with PP (paliperidone ER/PP HR 2.52; 95% CI 1.46 to 4.35; *p* < 0.001). Similarly, the ER placebo group had a higher risk of relapse than the PP placebo group. These placebo results are likely due to the shorter half-life of paliperidone ER compared with that of paliperidone LAI. As a consequence, the LAI placebo group continued to receive benefit from residual plasma concentrations of paliperidone for many weeks after the final injection of PP.

The LS mean change for the PANSS total score at end point was 3.5 points lower for PP (*p* = 0.025), despite a nearly fourfold longer period of observation for the PP group. Results of the PSP evaluation suggest that improved symptomatic outcome with PP translates to better maintenance of functioning.

Both PP and paliperidone ER were well tolerated. What appeared to be similar rates of AEs of interest were observed for the two formulations, and no additional safety concerns were associated with LAI versus oral paliperidone. Indeed, the numerical incidence rates favoured PP for all AEs other than weight gain despite the fourfold longer period of exposure for the PP group. The greater weight gain in the PP group may have been related to the longer period of exposure and increased time to gain weight.

This study has several limitations: (1) It was not possible to compare results from these studies by examining differences from placebo treatment because of the large differences in pharmacokinetic properties. The LAI formulation used in the stabilisation phase remained in the plasma for several months (median apparent t½ 25 to 49 days, depending on dose), whereas the oral formulation was cleared in days (median t½ 23 h). Thus, for several months after the start of the DB phase, the placebo group formerly on the LAI still was effectively on treatment and those in the placebo arm of the oral formulation study were not. (2) These studies used slightly different definitions of *relapse*; however, the most common reason for relapse in all groups was an increase in total PANSS of ≥25%. Subjects who met the additional criterion in the paliperidone ER study also met the other relapse criteria; therefore, no adjustment to the relapse definition was necessary. (3) The median duration of exposure in the DB phase was 171 days for the PP study versus 45 days for the paliperidone ER study. It is not possible to correct for differences in exposure, as exposure differences were due to trial design, different pharmacokinetic parameters of paliperidone ER and paliperidone palmitate and efficacy differences (both studies stopped early after interim analyses, and patients in the ER group relapsed sooner). Also, the impact on AEs is complex because the rate of occurrence of adverse events is not constant over time (AEs tend to occur more frequently early after exposure rather than late in the trial). (4) The combined run-in/transition/stabilisation phases were longer for the PP study than for the paliperidone ER study (33 weeks vs 14 weeks, respectively); therefore, subjects in the PP study had maintained stabilisation for a longer period. This may have resulted in a greater level of stability in the PP group at the time of randomisation. Additionally, the paliperidone ER study required subjects to experience an acute episode at study entry, whereas the PP study did not. These differences in inclusion criteria may have influenced the study results. (5) The dose range used in the paliperidone ER study was 3 to 15 mg/day, which is higher than the approved range (3 to 12 mg/day). In contrast, the allowable dose range in the PP study was 39 to 156 mg (25 to 100 mg eq), which roughly equates to 2 to 8 mg/day of paliperidone ER and is narrower than the approved range of 39 to 234 mg (25 to 150 mg eq). This difference in dose could potentially bias the results of the study in favour of paliperidone ER. (6) Higher proportions of African American subjects (19% vs 8%) and Asian subjects (16% vs 3%) and a lower proportion of other subjects (2% vs 30%) were treated with PP compared with subjects treated with paliperidone ER. It is unknown whether these racial differences may have affected the study results.

## Conclusions

This analysis supports maintenance of effect with the injectable compared with the oral formulation of paliperidone in patients with schizophrenia. This finding may be due to the adherence advantage of LAI over the oral agent. The prolonged release characteristics of PP may provide a longer protective advantage over paliperidone ER in subjects at risk for abrupt discontinuation. Despite the long-acting nature of PP, its AE profile appeared similar to that of paliperidone ER. Future studies are needed to confirm these findings.

## Abbreviations

AE: Adverse event; CGI-S: Clinical Global Impressions–Severity; DB: Double-blind; CI: Confidence interval; EPS: Extrapyramidal symptoms; ER: Extended release; HR: Hazard ratio; LAI: Long-acting injectable; LS,: Least squares; PANSS: Positive and Negative Syndrome Scale; PP: Paliperidone palmitate; PSP: Personal and Social Performance; t½: Terminal half-life; TEAE: Treatment-emergent adverse event.

## Competing interests

This work was funded by Janssen Scientific Affairs, LLC. MM, DJF and LA are employees of Janssen Scientific Affairs, LLC, and are Johnson & Johnson stockholders. BL, SG and IT are employees of Janssen Research & Development, LLC, and are Johnson & Johnson stockholders.

## Authors’ contributions

MM contributed to study conceptualisation and management, study design, data analysis and writing. DJF, BL and LA contributed to study design, data analysis and writing. SG contributed to study design, data analysis, data collection and writing. IT contributed to study design, data collection, writing and statistical analyses. All authors critically reviewed and revised the manuscript and approved the final manuscript.
